# Combination of probiotics and coccidiosis vaccine enhances protection against an *Eimeria* challenge

**DOI:** 10.1186/s13567-016-0397-y

**Published:** 2016-11-08

**Authors:** Miranda M. Ritzi, Wael Abdelrahman, Kobus van-Heerden, Michaela Mohnl, Nathaniel W. Barrett, Rami A. Dalloul

**Affiliations:** 1 Avian Immunobiology Laboratory, Department of Animal & Poultry Sciences, Virginia Tech, Blacksburg, VA USA; 2 Faculty of Veterinary Medicine, Suez Canal University, Ismailia, Egypt; 3 Ceva Animal Health, Guelph, Ontario, Canada; 4 BIOMIN Holding GmbH, Herzogenburg, Austria

## Abstract

Coccidiosis is endemic in the commercial broiler industry capable of inflicting devastating economic losses to poultry operations. Vaccines are relatively effective in controlling the disease; their efficacy could potentially be improved with concurrent use of probiotics as evaluated in this study using an *Eimeria* challenge. Day of hatch 400 Cobb-500 male broilers were assigned to one of four treatment groups including control (CON), vaccine-only gel application (VNC), probiotic-only gel application (NPC), and vaccine-plus-probiotic gel application (VPC). Birds were placed in floor pens (6 replicate pens/treatment, 16–17 birds/pen). NPC and VPC birds received the probiotics in the water on days 2–4, 8, 14–20, 22, 29, and 34–36. On day 15, birds were mildly challenged with 0.5 mL of a mixed oral inoculum of *Eimeria* sp. prepared with the coccidiosis vaccine at 10× the vaccination dose. Performance measurements were recorded on first day and weekly afterwards, and lesion scores were evaluated 6 days post-challenge. Overall, the probiotics and coccidiosis vaccine resulted in an enhanced protective effect against the challenge, with VPC birds exhibiting lower lesion scores in the duodenum than VNC or NPC birds. Birds in the VPC treatment also demonstrated higher weight gains during days 1–15, days 7–15, and days 21–28 when compared to the VNC birds. These results suggest that the combination of probiotics and coccidiosis vaccines could enhance performance and provide an additional protective effect against a mixed *Eimeria* challenge.

## Introduction

The practice of supplying food animals with sub-therapeutic doses of antibiotics to protect against coccidiosis infections and improve general intestinal health has recently been under scrutiny. The relatively recent ban of sub-therapeutic doses of certain antibiotics as feed additives in the European Union has led to a general decline in animal health with increased incidences of enteric conditions [1] known as dysbiosis. This outcome, as well as the threat of a domestic ban, has led researchers to explore the next promising alternatives including probiotics and potential combinations with live oocyst vaccines.

Although the primary function of the gastrointestinal tract is to digest and absorb nutrients, a well-balanced gut microbiota is crucial for optimal animal health and performance. The gastrointestinal tract also serves as a vital barrier preventing the entry of potentially harmful pathogens and other environmental antigens [2]. As the gut microbiota begins to establish within hours after the chick hatches, the earlier the introduction of nonpathogenic microorganisms, the more effective their establishment in the digestive tract [3, 4]. Also known as direct-fed microbials, probiotics are classified as live nonpathogenic microorganisms that are capable of maintaining a normal gut microbial population [5, 6]. Probiotics can help maintain a healthy balance of microorganisms through multiple modes of action including competitive exclusion, pathogen antagonism, and stimulation of the immune system [6, 7]. Probiotics may provide a potential alternative to the prophylactic use of drugs in food animals due to their studied abilities to reduce severity of enteric diseases and enhance performance in poultry [5, 8–10]. Probiotics can be composed of one or many strains of microbial species, with the more common ones belonging to the genera *Lactobacillus*, *Bifidobacterium*, *Enterococcus*, *Bacillus*, and *Pediococcus* [11].

Coccidiosis is endemic in the commercial broiler industry and inflicts devastating economic losses to poultry operations estimated to cost the industry about US $3 billion annually worldwide [12]. Coccidiosis is caused by development and reproduction of multiple species of the *Eimeria* protozoa, leading to impaired growth and feed utilization and predisposing birds to secondary infections. Anticoccidial compounds have been used to control coccidiosis, but *Eimeria* species have developed resistance to both chemical and ionophore drugs over time [13]. As such, the use of live vaccines to control coccidiosis has greatly increased. Vaccines provide an alternative for disease protection, and they ultimately help in reducing *Eimeria* resistance as they systematically replace resistant field strains and induce specific protective immunity by exposing the chicken’s immune system to *Eimeria* antigens [13–15]. Immunity is subsequently boosted and maintained by multiple re-infections caused by oocysts present in the litter due to shedding and ingestion [14]. Early and uniform administration of live oocysts of the vaccine results in a low level infection, necessary for immunity development. However, it can cause an early reduction in growth and may increase the chick’s susceptibility to secondary infections, such as necrotic enteritis [13, 15, 16]. The potential consequences of coccidiosis vaccine administration at a young age could be overcome by proper and uniform delivery of the vaccine, as well as the chick having a healthy intestinal tract colonized by a normal pathogen-free microbiota [13, 15]. Probiotics have the potential to enhance host defenses and affect the digestive microbiota positively, while protecting against colonization by harmful bacteria and maintaining intestinal integrity [13, 15, 17–19]. Based on these findings, probiotics may be able to attenuate the negative consequences of early vaccine administration. This study aimed to evaluate the combined protective effects of a probiotic product (PoultryStar, BIOMIN GmbH, Austria) containing *Enterococcus*, *Bifidobacterium*, *Pediococcus* and *Lactobacillus* species, and a coccidiosis vaccine (Immucox I, CEVA Santé Animale, Canada) containing *Eimeria acervulina*, *E*. *maxima*, *E*. *necatrix*, and *E*. *tenella* oocysts, against a coccidiosis challenge in broiler chickens.

## Materials and methods

### Birds and experimental treatments

The study performed was a 42-day grow-out with 400 Cobb-500 male broilers housed on randomized floor pens (furnished with ~10 cm of clean wood shavings, which remained throughout the study period), with 6 replicate pens per treatment and 16 or 17 birds per pen. On day of hatch (DOH), 100 birds were treated for each of the following four treatments at the hatchery: 1) control (CON), 2) vaccinated-only (VNC), 3) water-applied probiotic only (NPC), and 4) vaccinated and water-applied probiotic (VPC). VNC and VPC birds received Immucox I vaccine through gel droplet administration at the hatchery. Birds in NPC and VPC received probiotics via gel droplet application at the hatchery using a Desvac Gel Dispenser, as well as in the water intermittently through the course of the trial. The gel product was prepared using cold tap water and the dried gel component provided with the vaccine. Once water was added, the live oocyst vaccine, probiotic product, or both were added based on treatment. Then, the components were thoroughly mixed with a hand held mixer on low speed until the mixture was consistent and all probiotic product was dissolved. Once prepared, the mixture was applied using a commercial gel droplet applicator. The probiotics were administered in the water at 20 mg/bird per day on the first three days after placement, once a week, the week of *Eimeria* species challenge starting one day prior to inoculation, and one day before, the day of, and one day after a feed change. In summary, probiotics were administered a total of 17 time points, including days 2–4, 8, 14–20, 22, 29, and 34–36 (shown in Table 1). All birds received a basal diet ad libitum (Table 2). All animal protocols were approved and conducted under the guidelines of the Virginia Tech Institutional Animal Care and Use Committee.

**Table 1:** **Administration of probiotics (PoultryStar®) in water to appropriate treatments**

**Day**	1	2	3	4	…	9	…	14	15	16	17	18	19	20	…	22	…	29	…	34	35	36	…	42
Treatment																								
CON																								
VNC																								
NPC		*	*	*		*		*	*	*	*	*	*	*		*		*		*	*	*		
VPC		*	*	*		*		*	*	*	*	*	*	*		*		*		*	*	*		

* Indicates probiotics were included in the drinking water at 20 mg/bird in each pen.

CON: control, VNC: vaccine administration only, NPC: probiotic administration only, VPC: both vaccine and probiotic administration.

**Table 2:** **Composition of broiler diets during 3 growing phases**

Item	Starter (DOH to day 15)	Grower (days 15–35)	Finisher (days 35–42)
Ingredient, %			
Corn	60.55	65.63	69.90
Soybean meal	22.42	16.43	10.76
Distiller’s grain	7.00	8.00	9.00
Poultry by-product meal	5.00	5.00	4.00
Grease (yellow)	1.91	2.12	2.79
Dicalcium phosphate	1.15	0.90	0.78
l-Lysine	0.63	0.60	0.80
Limestone	0.58	0.54	0.70
DL-Methionine	0.18	0.30	0.80
Salt	0.27	0.17	0.16
l-Threonine	0.10	0.10	0.10
Southern States vitamin premix	0.10	0.10	0.10
Southern States trace mineral premix	0.10	0.10	0.10
Optiphos	0.01	0.01	0.01
Total	100.00	100.00	100.00
Calculated nutrient level			
ME, kcal/kg	3036.00	3102.00	3157.00
CP, %	21.00	19.00	17.00
Ca, %	0.90	0.80	0.76
Available P, %	0.45	0.40	0.35
Total P, %	0.71	0.64	0.57
Digestible Lys, %	1.50	1.33	1.32
Digestible Meth, %	0.50	0.60	1.06
Digestible Thr, %	0.89	0.81	0.71
Digestible Trp, %	0.22	0.19	0.16

### *Eimeria* challenge

On day 15 of age, birds were challenged via oral gavage with 0.5 mL of the Immucox I coccidiosis vaccine, providing 10× the vaccine dose given at hatch, initiating a mixed *Eimeria* challenge. On day 21 (6 days post infection), 18 birds per treatment were randomly selected and euthanized for scoring of lesions from intestinal *Eimeria* infection. Lesions in the duodenum, jejunum, ileum and ceca were scored by the method of Johnson and Reid [20] by personnel blinded to treatments based on scores ranging from 0 (no gross lesion) to 4 (most severe lesion). Excreta samples were collected from each pen on days 6–8 and 14 after challenge. For each pen, fresh excreta samples were collected from either side of the feeder, either side of the water supply, and from the center of the pen. Samples were kept in separate airtight plastic bags. Starting excreta weights were recorded for each sample for later calculations of oocysts per gram of excreta as previously described [17]. After homogenization, samples were stored at 4 °C until oocysts were counted microscopically after dilution using a McMaster counting chamber and expressed as oocysts per gram of excreta. Weekly litter samples were collected from each pen to assess moisture content. Five samples were taken from each pen once a week and stored in airtight plastic bags. The samples were transferred to paper bags and placed in a drying oven at 55 °C for 24 h, with both starting and final weights recorded. From days 15–24, excreta in each pen were evaluated and scored for bloody diarrhea as described by Youn and Noh [21].

### Performance

Pen and feed weights were taken on DOH, days 7, 15, 21, 28, 35 and 42. From these data, body weight (BW), body weight gain (BWG), feed intake (FI), and feed conversion ratio (FCR) were determined on a pen basis, and then averaged by treatment. Mortality was checked twice daily and feed consumption was corrected accordingly. One VNC pen was excluded from all calculations due to high mortality in the first week of the study due to undetermined but suspected metabolic conditions.

### Statistical analysis

Data were analyzed using the Fit Model platform in JMP Pro 10.0 (SAS Institute Inc., Cary, NC, USA). For performance measurements, oocyst shedding, and litter moisture analysis, the model included treatment with pen representing the experimental unit. Lesion score analysis was performed with treatment in the model with bird serving as the experimental unit. Differences among experimental treatments were tested using Tukey HSD following ANOVA. Values were considered statistically different at *P* ≤ 0.05. Results are reported as Least Square Means (LS means) with standard errors of the mean (SEM).

## Results

### *Eimeria* challenge

#### Lesion scores

**Figure 1:**
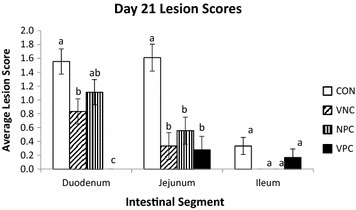
**Effect of administration of probiotics (PoultryStar) and coccidiosis vaccine (Immucox I) on day 21 lesion scores in the duodenum, jejunum, and ileum of Cobb-500 male broiler challenged with** ***Eimeria*** **species on day 15.** Data are presented as Least Square Mean ± SEM; bars lacking a common letter differ significantly. CON: control; VNC: vaccine administration only; NPC: probiotic administration only, VPC: both vaccine and probiotic administration.

On day 21, a significant effect of treatment (*P* < 0.0001) was noted in lesion scores in the duodenum, the site of *E*. *acervulina* infection, as presented in Figure 1. The CON birds had significantly higher lesion scores than VNC and VPC birds (*P* < 0.0001). No lesions were observed in the duodenum of VPC birds, resulting in VPC being significantly different from all other treatments. The jejunum is one segment prone to damage from *E*. *maxima* and *E*. *necatrix*; the CON birds exhibited significantly higher lesions in the jejunum than all other treatments (*P* < 0.0001). No lesions were observed in the ileum of VNC or NPC birds on day 21. The average lesion scores in the ileum for CON and VPC birds were low, resulting in no significant differences seen among treatments. In addition, no lesions were observed in the ceca, the site of *E*. *tenella* infection, in any treatment (data not shown as all scores were effectively zero).

#### Litter moisture

**Figure 2:**
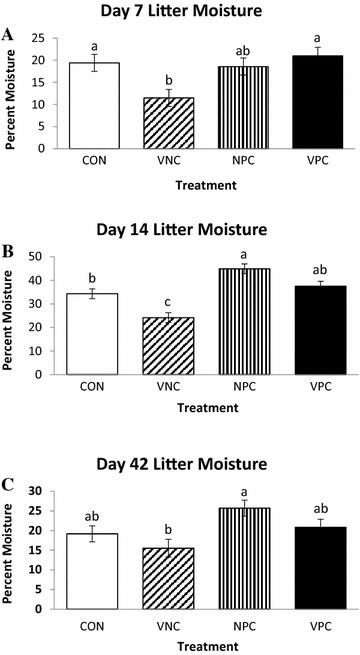
**Effect of administration of probiotics (PoultryStar) and coccidiosis vaccine (Immucox I) on litter moisture on days 7 (A), 14 (B), and 42 (C) in pens of Cobb-500 male broiler chickens.** Data are presented as Least Square Mean ± SEM; bars lacking a common letter differ significantly. CON: control; VNC: vaccine administration only; NPC: probiotic administration only, VPC: both vaccine and probiotic administration.

Figure 2A represents the significant effect of treatment (*P* = 0.0119) on litter moisture noted on day 7. The litter present in the VNC pens had significantly less moisture when compared to CON and VPC pens. Litter moisture also demonstrated a significant effect of treatment on day 14 (Figure 2B) where VNC pens had lower (*P* < 0.0001) litter moisture when compared to all other treatments. In addition, NPC pens had higher percent moisture of the litter when compared to the CON pens (*P* < 0.0001). At the end of the trial, a significant effect of treatment (*P* = 0.0245) was noted on percent moisture of the litter. Shown in Figure 2C, NPC pens had significantly higher percent moisture than VNC pens only.

#### Bloody diarrhea scores

No significant differences among treatments were noted regarding the presence of bloody diarrhea from days 15–24 (data not shown).

### Performance

Results for performance parameters are summarized in Table 3.

**Table 3:** **Effect of administration of probiotics (PoultryStar) and coccidiosis vaccine (Immucox I) on performance**

Variable	Treatment^a^				SEM	*P* value
	CON	VNC	NPC	VPC		
DOH-day 7						
DOH BW, g	37.41^A^	37.37^A^	38.23^A^	37.39^A^	0.25	0.6090
Day 7 BW, g	150.85^A^	142.37^A^	154.17^A^	150.50^A^	3.31	0.1039
BWG, g	113.44^A^	105.00^A^	116.33^A^	113.1^A^	3.28	0.1202
FI, g/bird/day	30.01^A^	28.14^A^	37.62^A^	37.61^A^	3.31	0.1111
FCR	1.86^A^	1.85^A^	2.27^A^	2.33^A^	0.21	0.2525
Days 7–15						
Day 15 BW, g	362.93^AB^	341.23^B^	379.42^A^	379.22^A^	7.88	0.0078
BWG, g	212.08^AB^	198.87^B^	225.25^AB^	228.72^A^	7.08	0.0289
FI, g/bird/day	46.82^B^	59.89^A^	48.38^AB^	49.03^AB^	3.31	0.0425
FCR	1.55^A^	2.14^B^	1.50^A^	1.51^A^	0.13	0.0074
Days 15–21						
Day 21 BW, g	621.35^AB^	583.33^B^	709.36^A^	610.90^AB^	30.23	0.0434
BWG, g	258.41^A^	242.10^A^	329.94^A^	231.68^A^	28.10	0.0898
FI, g/bird/day	78.70^A^	88.16^A^	86.77^A^	82.44^A^	6.08	0.6864
FCR	1.90^A^	2.02^A^	1.52^A^	3.34^A^	0.84	0.4629
Days 21–28						
Day 28 BW, g	1292.32^A^	1121.92^B^	1352.72^A^	1394.23^A^	38.37	0.0008
BWG, g	670.97^AB^	531.92^B^	643.36^AB^	783.33^A^	38.68	0.0032
FI, g/bird/day	296.79^A^	292.01^A^	357.28^A^	351.49^A^	28.96	0.2824
FCR	3.07^A^	4.14^A^	4.10^A^	3.13^A^	0.52	0.3192
Days 28–35						
Day 35 BW, g	1935.92^A^	1847.17^A^	2025.32^A^	2018.63^A^	47.01	0.0648
BWG, g	643.61^A^	725.25^A^	672.61^A^	624.40^A^	43.82	0.4593
FI, g/bird/day	150.40^A^	201.94^A^	155.85^A^	187.38^A^	27.80	0.5428
FCR	1.74^A^	2.01^A^	1.67^A^	2.06^A^	0.32	0.7782
Days 35–42						
Day 42 BW, g	2799.75^A^	2749.44^A^	2874.86^A^	2835.99^A^	56.11	0.4954
BWG, g	863.83^A^	902.28^A^	849.54^A^	817.36^A^	47.24	0.6835
FI, g/bird/day	183.59^A^	176.74^A^	188.32^A^	179.27^A^	6.29	0.6151
FCR	1.50^A^	1.39^A^	1.56^A^	1.54^A^	0.05	0.1237
DOH-day 15						
BWG, g	325.52^AB^	303.86^B^	341.58^A^	341.82^A^	7.84	0.0079
FI, g/bird/day	38.62^A^	41.17^A^	43.10^A^	43.50^A^	2.68	0.5680
FCR	1.84^B^	2.44^A^	1.93^AB^	1.93^AB^	0.15	0.0358
Days 15–35						
BWG, g	1572.99^A^	1497.69^A^	1645.91^A^	1639.41^A^	47.24	0.1589
FI, g/bird/day	164.29^A^	183.29^A^	189.41^A^	192.51^A^	11.64	0.3432
FCR	2.11^A^	2.08^A^	2.32^A^	2.76^A^	0.35	0.5153
DOH-day 42						
BWG, g	2762.33^A^	2712.07^A^	2837.03^A^	2798.59^A^	56.22	0.4999
FI, g/bird/day	107.01^A^	113.48^A^	122.96^A^	120.47^A^	4.40	0.0775
FCR	1.63^A^	1.76^A^	1.82^A^	1.81^A^	0.06	0.1383

^a^CON: control, VNC: vaccine administration only, NPC: probiotic administration only, VPC: vaccine and probiotic administration, DOH: day of hatch.

^A–B^Means within rows that do not have a common superscript differ significantly (*P* ≤ 0.05).

#### Body weight (BW)

A significant effect of treatment (*P* = 0.00078) was seen on day 15, with NPC and VPC birds demonstrating significantly higher average BW than VNC birds, while CON birds were comparable to all other treatments. A significant effect of treatment was also seen on day 21, with NPC birds having greater BW than the VNC birds (*P* = 0.0434). However, the average body weights of CON and VPC birds did not differ significantly from each other or the other treatments on day 21. Average BW on day 28 showed a significant effect of treatment, with VNC birds weighing less than all other treatments (*P* = 0.0008).

#### Body weight gain (BWG)

A significant effect of treatment was seen from DOH-day 15 regarding BWG, with probiotic-treated birds (NPC and VPC) gaining more weight than VNC birds (*P* = 0.0079). Within the starter phase, from days 7–15, VPC birds gained more weight than VNC birds only (*P* = 0.0289). The same trend was observed from days 21–28, where VPC birds had significantly higher BWG than VNC birds, with CON and NPC birds demonstrating comparable weight gains (*P* = 0.0032).

#### Feed intake (FI)

A significant effect of treatment was seen from days 7–15 regarding FI, with VNC birds consuming more feed per day than CON birds (*P* = 0.0425).

#### Feed conversion ratio (FCR)

For the period prior to challenge (DOH-day 15), VNC birds had significantly higher FCR than CON birds (*P* = 0.0358). From days 7–15, VNC birds demonstrated higher FCR than all other treatments (*P* = 0.0074).

## Discussion

In this study, the combined protective effects of probiotics and coccidiosis vaccine in the event of an *Eimeria* challenge were evaluated. Birds in the combined vaccine/probiotic treatment group had less severe duodenal lesion scores than all other treatments. Further, CON birds had lesions of greater severity than VNC birds in the duodenum, as well as lesion scores significantly greater than all other treatments in the jejunum. These findings suggest that probiotic supplementation, vaccine administration, and a combination of both help prevent damage to the intestine from coccidia. Similarly, Lee et al. [22] reported that birds given a *Bacillus*-based direct-fed microbial had significantly lower lesion scores in the gut than birds given the non-supplemented diet following an *E*. *maxima* challenge. Studies investigating necrotic enteritis (NE) in broilers found birds given two different blends of direct-fed microbials had significantly reduced intestinal lesions due to NE than birds in the positive control [23]. Additionally, birds that received just a coccidiosis vaccine had less severe lesions in the upper and middle intestinal segments following challenge with three *Eimeria* species when compared to non-vaccinated birds that were fed therapeutic levels of an ionophore anticoccidial [16].

A second study found that birds given one of three different coccidiosis vaccine doses had less severe lesion scores than the positive non-vaccinated control [16]. Less severe lesion scores are indicative of less damage to the intestinal epithelium, leading to infected birds having a greater chance of recovery from disease. Numerous studies have found that probiotic supplementation leads to significant reductions in numbers of other intracellular pathogens [24, 25], which could prove to be exceptionally beneficial to the bird, as some microorganisms such as *Clostridium* and *Salmonella* may exacerbate *Eimeria* infections and vice versa [26]. Ultimately, the reduction in the presence of intracellular pathogens is indicative of a healthier intestine, with minimal damage done to the epithelium. An intact intestinal epithelium serves as the vital barrier preventing entry of potential pathogens and results in proper nutrient absorption and utilization, leading to optimal health and performance of the bird.

The presence of oocysts in the litter and excreta after vaccination is crucial in vaccinated flocks, as it indicates proper vaccine uptake. Vaccine efficacy is dependent upon the infectivity and fecundity of oocysts, since protective immunity is induced after two to three consecutive infections [14, 27, 28]. The re-infections are initiated by recycling of initially low doses of oocysts which result in gradual buildup and maintenance of immunity, and such recycling can be impacted by a number of factors including litter moisture content [29, 30]. The percent moisture in VNC pens was significantly lower on day 7, day 14, and day 42. Numerous studies have found that oocysts sporulate better in drier litter conditions, suggesting maximum sporulation rate and litter moisture are indirectly correlated [14, 26, 31]. As the infective state of the *Eimeria* life cycle is the sporulated oocyst, birds on litter with lower percent moisture could be introduced to a greater number of infective oocysts, leading to a heavier infection. Bloody diarrhea is commonly associated with *E*. *tenella*, which was present in the challenge inoculum. However, as no significant differences were noted, the dose of *E*. *tenella* present in the fresh vaccine may not have been sufficient to cause extensive damage to the site of infection, as confirmed by the absence of lesions in the ceca.

Vaccine-plus-probiotic gel application birds demonstrated significantly greater weight gains from DOH to day 15 when compared to VNC birds, suggesting the addition of probiotics helped the birds counter the reduction in growth associated with administration of coccidiosis live vaccines. However, the lack of a significant difference among treatments regarding BW at the end of the trial indicates VNC birds experienced compensatory growth following the initial setback from vaccination. These results coincide with the findings of Li et al. [16], in which a “reaction” caused by some doses of vaccine resulted in delayed growth and coccidial lesions during the two weeks following vaccination. However, the vaccinated birds exhibited a compensatory weight gain that brought them to weights almost equal to the unchallenged control by 5–6 weeks of age [16]. The effect of *Eimeria* challenge on BW and BWG is not surprising, as coccidial infections are known to cause damage to the intestinal mucosa and enterocytes during the progression of their lifecycle. Significant damage causes nutrient malabsorption and subsequent reduced performance. Furthermore, parasitic infections result in nutrient resource allocation shifting from growth to immune response, which can also lead to noticeable differences in growth [15, 29]. Numerous studies investigating probiotics as dietary additives in poultry have resulted in varying effects of those probiotics on performance. Some reported that probiotic supplementation in the diet can improve BWG and FCR in chickens [8, 32–34], while others found no significant benefit to probiotic addition [35, 36]. These differences could be due to a variety of factors that can alter the efficacy of a probiotic such as strain(s) of bacteria utilized, composition and viability of the probiotic bacteria, and the preparation methods. Further, other factors may include probiotic dosage, method and/or frequency of application, overall diet, condition and age of the birds, presence or absence of disease challenges, potential drug interactions, as well as environmental stress factors such as temperature and stocking density [5, 37].

Live vaccines offer a route of protection that circumvents the issue of developing drug-resistant coccidia [14, 26]. As vaccination induces protective immunity due to exposure of the immune system to *Eimeria* antigens, the birds may respond with a strong immune response more quickly to a field strain *Eimeria* infection [13, 28]. In conclusion, the administration of probiotics (PoultryStar) and coccidiosis vaccine (Immucox I) resulted in an enhanced protective effect against *Eimeria acervulina* and *E*. *maxima* challenge. The results of this study suggest that the combination of probiotics and coccidiosis vaccine can, when compared to untreated controls, result in better performance and intestinal response. Early establishment of beneficial microbiota by probiotics can inhibit pathogens thus potentiating a protective effect and enhancing host resistance to infection while reducing the need for prophylactic drug usage [5, 19, 38]. As PoultryStar is a product that contains multiple probiotic species of bacteria, there is a greater potential that such probiotics can be active in a wider range of conditions, similar to other multi-strain probiotics, resulting in greater efficacy [3, 39]. Probiotics may enhance host defenses and improve vaccine response as a result of the influence of beneficial bacteria on host immunity and intestinal integrity against enteric pathogens [13, 17]. Together, probiotics and coccidiosis vaccines can benefit performance and provide an augmented protective effect in the event of an *Eimeria* challenge.
